# Circular RNA coiled-coil domain containing 66 regulates malignant development of papillary thyroid carcinoma by upregulating La ribonucleoprotein 1 *via* the sponge effect on miR-129-5p

**DOI:** 10.1080/21655979.2022.2036304

**Published:** 2022-03-09

**Authors:** Peipei Li, Junhui Chen, Jun Zou, Wei Zhu, Yan Zang, Hongwu Li

**Affiliations:** a Department of Otorhinolaryngology Head and Neck Surgery, The Fourth Affiliated Hospital of Anhui Medical University, Hefei, China; b Department of Neurosurgery, Wuxi Clinical Medical School of Anhui Medical University, 904th Hospital of PLA(Wuxi Taihu Hospital), Wuxi, China; c Department of Otolaryngology, Wuxi No. 5 People’s Hospital, Wuxi, China

**Keywords:** Papillary thyroid carcinoma, circular RNA, circ-CCDC66, miR-129-5p, LARP1

## Abstract

Circular RNAs (circRNAs) play vital roles in the development and progression of various diseases. CircRNA coiled-coil domain containing 66 (circ-CCDC66) has been reported to be involved in several cancers, but its biological function and underlying mechanism in papillary thyroid carcinoma (PTC) remain unclear. We detected the relative expression level of circ-CCDC66 in PTC specimens and cell lines using real-time reverse transcription PCR. In addition, EdU assay, transwell assay, and xenograft analysis were performed to measure the effect of circ-CCDC66 on the proliferative, migratory, and invasive capacities of PTC cells. We also investigated the potential mechanism of circ-CCDC66 by bioinformatics analysis, RNA immunoprecipitation, and dual-luciferase reporter assay. We observed that circ-CCDC66 expression was upregulated in PTC specimens and cell lines and was correlated with poor clinical characteristics of PTC patients. Moreover, *in vitro* experiments demonstrated that knockdown of circ-CCDC66 markedly suppressed the proliferative, migratory, and invasive capacities of PTC cells. Mechanistically, miR-129-5p was a target gene of circ-CCDC66 and was downregulated in PTC tissues. LARP1, a downstream target of miR-129-5p, was upregulated in PTC tissues. In addition, we confirmed that inhibition of circ-CCDC66 could repress xenograft tumor growth. Circ-CCDC66 promoted PTC proliferation, migration, invasion, and tumor growth by sponging miR-129-5p and promoting LARP1 expression.

## Graphical Abstract

**Figure:**
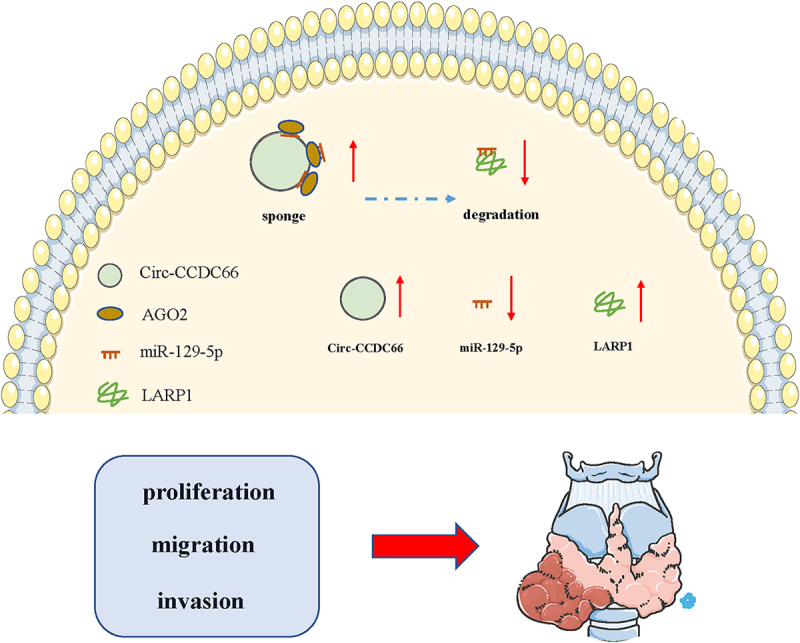

## 1. Introduction

Thyroid carcinoma (TC) is the most common malignant tumor of the endocrine system, with an incidence increasing by approximately 3% every year [1,2]. TC mortality rates are generally low; however, TC mortality rates have increased by approximately 1.1% per year over the past 40 years [3,4]. Papillary TC (PTC) accounts for 85% of all TC cases, affecting all age groups [5]. It is a commonly diagnosed TC with relatively low malignancy rates [6,7]. In recent years, the incidence of PTC has increased annually due to environmental, genetic, and hormonal factors. While most PTC cases have good prognosis, some PTC cases are invasive, leading to the involvement of adjacent organs, extraglandular invasion, or lymph node metastasis at the early stage. As a result, surgical resection rate is markedly reduced, resulting in poor prognosis [8,9]. Therefore, an in-depth understanding of the molecular mechanisms underlying invasiveness and metastasis of PTC is urgently needed for better assessment of invasive risk, and clinical guidance. Many studies have shown that certain proteins are responsible for the biological behaviors of PTC cells and can be utilized as therapeutic targets [10,11]. It is generally believed that invasiveness, local recurrence, and distant metastasis are the main factors affecting cancer prognosis. However, the precise pathogenesis attributed to the invasiveness and metastasis of PTC is not fully understood.

Circular RNAs (circRNAs) are a novel type of endogenous non-coding RNA (ncRNA) characterized by a closed covalent loop in the absence of a 5ʹ cap and 3ʹ poly (A) tail. They are abundantly, robustly, and conservatively expressed [12–14]. According to their sources, circRNAs are classified as ecRNAs, elciRNAs, and ciRNAs, and the former accounts for the majority of circRNA [15–17]. CircRNAs have diverse biological functions in cancer development, such as serving as competing endogenous RNAs (ceRNAs), which competitively bind microRNAs (miRNAs) to further affect the biological functions of their target genes, which is the most reported mechanism of circRNAs in cancer development [18–20]. In brief, miRNAs are single-chain small-molecular RNAs that can inhibit translation or induce degradation of target genes by recognizing and binding to the 3ʹ UTR. Moreover, miRNAs have been reported to be involved in various cancers [21]. miR-335 can inhibit the aggressive phenotypes of ovarian cancer cells by inhibiting COL11A1 expression [22]. miR-210-3p can inhibit pancreatic tumor growth and proliferation by targeting MUC4 [23]. miR-129-5p has been reported to act as a tumor suppressor in various cancers, including gastric cancer [24], colon cancer [25], hepatocellular cancer [26], prostate cancer [27], and breast cancer [28]. CircRNAs also exert a ‘sponge’ effect on proteins to alter their subcellular distribution, mediate transcription of parent genes, and promote protein–protein interaction [29,30]. In addition, circRNAs have translational potential, as some circRNAs contain an internal ribosome entry site (IRES) and open reading frame (ORF), and are capable of intracellularly translating proteins [31,32]. It has also been reported that circRNAs (elciRNAs and ciRNAs) can regulate the expression of parent genes by mediating the transcription activities of RNA Polymerase II and other transcription factors [33,34]. Collectively, circRNAs are functional mediators involved in cancer cell behavior through multiple mechanisms.

CircRNA coiled-coil domain containing 66 (circ-CCDC66) has been identified as a potential therapeutic target in cancer. It has been reported that circ-CCDC66 is upregulated in colorectal cancer (CRC) cases and affects cancer development by mediating certain oncogenes. Knockdown of circ-CCDC66 inhibits the growth and invasiveness of CRC in xenograft and *in situ* mouse models [35]. Circ-CCDC66 has been reported to be upregulated not only in renal cancer cell lines but also in tumor stem cell spheres, and enhances the enrichment of tumor stem cells [36]. Circ-CCDC66 can promote proliferation, invasion, and migration of glioma cells by suppressing miR-320a and promoting FOXM1 expression [37]. Circ-CCDC66 can upregulate REXO1 expression to aggravate cervical cancer progression by binding to miR-452-5p [38]. Moreover, many studies have confirmed that corresponding preclinical research can be carried out for some key genes, providing a new perspective for tumor prevention and treatment [39–43]. However, the exact function and clinical potential of circ-CCDC66 in PTC remain to be explored.

In the present study, circ-CCDC66 expression was found to be upregulated in PTC specimens and cell lines. Knockdown of circ-CCDC66 markedly suppressed the proliferative, migratory, and invasive capacities of PTC cells. Furthermore, functional experiments demonstrated that circ-CCDC66 promoted the development of PTC by upregulating La-related protein 1 (LARP1) by exerting a ‘sponge’ effect on miR-129-5p.

## 2. Materials and methods

### 2.1. Participants and specimens

This study was approved by the Ethics Committee of the Fourth Affiliated Hospital of Anhui Medical University (Hefei, China) and conducted in accordance with the Declaration of Helsinki. All participants provided written informed consent. A total of 60 PTC and paired paracancerous specimens were collected, frozen in liquid nitrogen, and stored at −80°C. Tumor staging was assessed based on the guidelines proposed by the Union for International Cancer Control. Clinical data was recorded, and none of the recruited patients underwent preoperative radiotherapy or chemotherapy, or had other types of malignancies.

### 2.2. Cell culture

The human thyroid cell line Nttry-ori-3-1 and PTC cell lines IHH4, BCPAP, K1 and TPC-1 were obtained from ATCC (Manassas, VA, USA). All cells were cultured in RPMI-1640 (Invitrogen, CA, USA) supplemented with 10% fetal bovine serum (FBS) (Invitrogen), 100 U/mL penicillin, and 0.1 mg/mL streptomycin in a humidified 5% CO_2_ incubator at 37°C and passaged at 80–90% confluence. The identity of the cells used in the experiment was confirmed using short tandem repeat analysis. All cell lines were tested for Mycoplasma every 3 months.

### 2.3. Real-time reverse transcription PCR (qRT-PCR)

Total cellular RNA was extracted using TRIzol (Invitrogen) and stored at −80°C until further analysis. The Prime Script RT Reagent Kit (Takara, Dalian, China) was used to obtain cDNA from the reverse transcription of RNAs (500 ng). qRT-PCR was performed using 2 µL cDNA as the template, 1 µL each of forward and reverse primers and the SYBR Green qPCR Mix kit. The PCR reaction conditions were as follows initial denaturation at 95°C for 10 min; 40 cycles of denaturation at 95°C for 30 s, annealing at 60°C for 30s, and extension at 72°C for 30 s; final extension at 72°C for 10 min. qRT-PCR was performed on the ABI7900 fluorescent PCR instrument (Applied Biosystems, Waltham, MA, USA). U6 and GAPDH were used as the internal reference genes. The relative mRNA levels were calculated using the 2^−ΔΔCT^ method [44]. Sequences of primers used for qRT-PCR are listed in Table 1.

**Table 1.** Sequences of primers for qRT-PCR

Name		Sequence
Circ-CCDC66	Forward	5’- TCTCTTGGACCCAGCTCAG −3’
	Reverse	5’- TGAATCAAAGTGCATTGCCC −3’
miR-129-5p	Forward	5’- CGGCGGTTTTTTGCGGTCTGGGCT −3’
	Reverse	5’- AGCCCAGACCGCAAAAAACCGCCG −3’
LARP1	Forward	5’ – GCAACCTAAAGACACTAC-3’
	Reverse	5’-GTGCAGGGTCCGAGGT-3’
GAPDH	Forward	5’-GCACCGTCAAGGCTGAGAAC-3’
	Reverse	5’-GGATCTCGCTCCTGGAAGATG-3’
U6	Forward	5’- GCTTCGGCAGCACATATACTAAAAT-3’
	Reverse	5’- CGCTTCACGAATTTGCGTGTCAT −3’

### 2.4. Actinomycin D assay

The actinomycin D assay was performed as described previously [45]. K1 and TPC-1 cells were exposed to 2 μg/mL actinomycin D (Sigma-Aldrich, St. Louis, MO, USA). qRT-PCR was then performed as described above to detect relative levels of circ-CCDC66 and mRNA levels of CCDC66.

### 2.5. RNase R assay

The RNase R assay was performed as described previously [45]. Briefly, 2 mg RNA with or without 5 U/μg RNase R (Epicenter Technologies, Madison, WI, USA) was incubated for 30 min in a water bath at 37°C. Then, RNeasy MinElute kit (Qiagen, Hilden, Germany) was used to purify the sample, and qRT-PCR was performed as described above.

### 2.6. Cell transfection

Small interfering (si) RNAs targeting circ-CCDC66 (si-circ-CCDC66#1 and #2), LARP1 siRNA, and corresponding negative controls were synthesized by GenePharma (Shanghai, China). The mimic/inhibitor miR-129-5p and the negative control were also obtained from GenePharma. Short hairpin (sh) RNA targeting circ-CCDC66 (sh-circ-CCDC66) and sh-NC were designed and purchased from Genecopoeia (Guangzhou, China). The lentiviral vector for circ-CCDC66 was purchased from GeneCreate Biological Engineering (Wuhan, China). Cell transfection was performed using Lipofectamine 3000 (Invitrogen) according to the manufacturer’s instructions, and the transfection efficacy was tested at 48 h. The sequence of siRNAs used was: si-circ-CCDC66 #1, sense: 5ʹ-AUUUUCUUUGCAGUUCUUGUU-3ʹ, antisense: 5ʹ-CAAGAACUGCAAAGAAAAUGG-3ʹ; si-circ-CCDC66 #2, sense: 5ʹ-AAUAUAUAAUUUUUUCCUCUA-3ʹ, antisense: 5ʹ-GAGGAAAAAAUUAUAUAUUCA-3ʹ; LARP1 siRNA, sense: 5ʹ-AUAGUUAAAACUUCAGAACAA-3ʹ, antisense: 5ʹ- GUUCUGAAGUUUUAACUAUUA-3ʹ. Transfection ef-ficiency of more than 70% was considered as an effective transfection.

### 2.7. Proliferation assay

#### Cell counting kit-8 (CCK-8) assay

CCK-8 assay was performed as described previously [46]. Briefly, approximately 2 × 10^3^ transfected cells were seeded per well in a 96-well plate and cultivated for 0, 24, 48, and 72 h, respectively. The cells were exposed to CCK-8 solution for 2 h, followed by detection at 450 nm using a microplate reader (BioTek Instruments, Winooski, VT, USA).

#### EdU assay

EdU assay was performed as described previously [47]. Briefly, 3 × 10^3^ transfected cells were seeded per well in a 96-well plate and exposed to 50 mM EdU (RiboBio, Guangzhou, China) for 24 h. Cells were then fixed using 4% methanol and permeabilized with Triton X-100. The cells were then cultivated in EdU mixed buffer and counterstained with DAPI. EdU-positive and DAPI-positive cells in five random fields per well were captured under a fluorescence microscope.

### 2.8. Transwell assay

Transwell assay was performed as described previously [47]. Transwell inserts (8 μm, Corning, Corning, NY, USA) were placed in a 24-well plate. The upper insert was pre-coated with 100 μg Matrigel, and then 4 × 10^4^ cells suspended in FBS-free medium were seeded in the upper chamber, and 500 μL of medium containing 10% FBS was added to the bottom chamber. Cells were allowed to penetrate for 24 h, and those in the bottom chamber were fixed with methanol for 15 min and stained with crystal violet for 20 min for visualization. Invasive cells were quantified by capturing five random fields per well (×200). Migratory cells were similarly measured in transwell inserts without pre-coating with Matrigel. The number of migratory or invaded cells was determined using ImageJ v1.5.

### 2.9. Subcellular fractionation

Subcellular fractionation was performed as described previously [48]. Briefly, approximately 4 × 10^4^ cells were transferred to a 1.5 ml EP tube and lysed in RLA on ice for 20 min, followed by centrifugation at 3,000 rpm for 15 min. The supernatant was collected as the cytoplasmic fraction. Next, the precipitant was washed in RLA three times and induced in RIPA on ice for 20 min, with 30 s vortex oscillation at 5 min intervals. The mixture was then centrifuged at 12,500 rpm for 15 min, and the supernatant was collected as the nuclear fraction.

### 2.10. Dual-luciferase reporter assay

Dual-luciferase reporter assay was performed as described previously [49]. The wild-type (WT) luciferase reporter vectors circ-CCDC66-WT and LARP1 3ʹ UTR-WT were constructed by inserting the sequence of circ-CCDC66 or LARP1 3 UTR containing miR-129-5p binding sites into the pmir-GLO vector. The mutant (MUT) circ-CCDC66-MUT and LARP1 3 UTR-MUT were constructed by mutating the complementary sites (CGTTTTT) of miR-136-5p to (GTTTTT). MUT or WT circ-CCDC66 (or LARP1) luciferase vector was co-transfected in K1 and TPC-1 cells with either negative control or miR-129-5p mimics for 48 h. Cells were processed using the dual-luciferase reporter assay kit (Promega, Madison, WI, USA) to measure relative luciferase activity.

### 2.11. Western blot

Western blot was performed as described previously [50]. Briefly, PTC cells were lysed in PMSF on ice to extract protein and the protein concentrations were measured using BCA protein kit (Beyotime, Shanghai, China). Protein samples (30 μg) were denatured at 100°C and then separated by 10% SDS-PAGE, and loaded onto PVDF membranes, which were cut into pieces according to the molecular size of the protein of interest. Membranes were immunoblotted with primary and secondary antibodies and exposed to analyze the protein bands. The primary antibodies used were as follows: LARP1 (ab86359, 1:3000, Abcam, Cambridge, UK) and GAPDH (ab8245, 1:1000, Abcam).

### 2.12. Immunohistochemistry (IHC)

IHC was performed as described previously [51]. Briefly, the PTC tissues were heated in citrate buffer and cooled to room temperature for antigen retrieval. The specimens were then incubated with LARP1 antibody (ab86359, 1:200, Abcam) overnight at 4°C, followed by incubation with a secondary antibody at room temperature for 2 h. The specimens were further stained with streptavidin-biotin-peroxidase reagents and fixed on gelatin-coated glass slides. Then, the average number of LARP1-positive cells was calculated.

### 2.13. Tumor xenograft assay

BALB/c male nude mice (4 weeks old) were purchased from Vital River Laboratory Animal Technology (Beijing, China). Tumor xenograft assay was performed as described previously [52]. Briefly, 5 × 10^6^ TPC-1 cells were stably transfected with lentivirus-carrying sh-circ-CCDC66 or sh-NC and subcutaneously injected into the right flank of nude mice. The volume (length × width^2^ × 0.5) of the tumor was calculated on days 0, 5, 10, 15, 20, and 25. The weight of the tumor was counted the 25th day. All procedures involving animals were approved by the Animal Care and Use Committee of the Fourth Affiliated Hospital of Anhui Medical University.

### 2.14. Statistical analysis

All experiments were performed three times. The GraphPad Prism 6 (GraphPad Software Inc., San Diego, CA, USA) and SPSS 22.0 (IBM, Armonk, NY, USA) were used to process data that were expressed as mean ± standard deviation. Differences between groups were compared using Student’s *t-test* and one-way ANOVA. Pearson correlation tests were conducted to assess the correlation between the genes. Kaplan–Meier curves were used for survival analysis. Statistical significance was set at p < 0.05.

## 3. Results

This study aimed to demonstrate the expression level, biological role, and potential mechanism of circ-CCDC66 in PTC. We demonstrated that circ-CCDC66 may play an essential role in the progression of PTC. Our results indicated that circ-CCDC66 expression levels were markedly increased in PTC tissues and cell lines, and circ-CCDC66 could sponge miR-129-5p to promote LARP1 expression, which can potentially accelerate malignant progression of PTC.

### 3.1. Circ-CCDC66 is upregulated and correlated with poor prognosis in PTC

**Figure 1.**
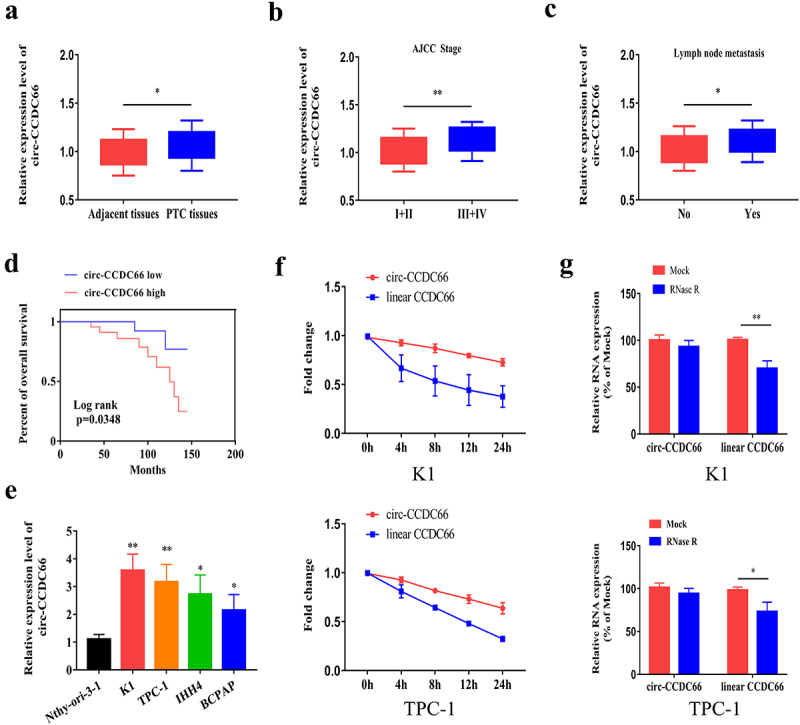
Circ-CCDC66 is upregulated in PTC and correlated to poor prognosis.

To explore the role of circ-CCDC66 in PTC, we detected relative levels of circ-CCDC66 by qRT-PCR in PTC samples and observed that circ-CCDC66 was highly expressed in PTC samples compared to the controls (Figure 1a). We further analyzed the correlation between circ-CCDC66 and the clinical data of patients with PTC. High levels of circ-CCDC66 were closely correlated with AJCC staging and lymphatic metastasis of PTC (Figure 1b-C–C). Survival curves were depicted using the Kaplan–Meier method based on the follow-up data of patients with PTC. As shown in Figure 1d, high levels of CCDC66 were predictive of poor prognosis in patients with PTC. We also analyzed the relationship between the expression levels of circ-CCDC66 and clinical pathological features. As shown in Table 2, the expression level of circ-CCDC66 was significantly associated with tumor size, TNM stage, and lymph node metastasis in patients with PTC. Additionally, circ-CCDC66 was upregulated in PTC cell lines compared to the normal thyroid cell line (Figure 1e). Among them, the expression of circ-CCDC66 was most significantly increased in K1 and TPC-1 cells, so we selected these two cell lines for *in vitro* experiments. Based on the features of stably expressed circRNAs, we examined the stability of circ-CCDC66 by exposing PTC cells to actinomycin D. Compared with the half-life of linear CCDC66 mRNA (<8 h), the half-life of circ-CCDC66 was longer than 24 h, confirming the stability of circ-CCDC66 (Figure 1f). Consistent with these findings, RNase R treatment did not influence the expression level of circ-CCDC66, whereas linear CCDC66 mRNA was markedly degraded (Figure 1g).

**Table 2.** Relationship between circ-CCDC66 expression and the clinical pathological characteristics of PTC patients (n = 60)

Clinic pathological features		NO. of cases	Circ-CCDC66 (n, %)		*p* – value
			Low	High	
Gender	Male	32	15	17	*P* > 0.05
	Female	28	13	15	
Age	≤ 55	40	18	22	*P* > 0.05
	> 55	20	8	12	
Extra thyroidal extension	Negative	19	8	11	*P* > 0.05
	Positive	41	16	25	
Tumor size	≤1	42	28	14	*P* = 0.0099
	>1	18	5	13	
TNM stage	I/II	34	22	12	*P* = 0.0362
	III/IV	26	9	17	
Lymph node metastasis	Negative	27	19	8	*P* = 0.0089
	Positive	33	11	22	
Nodular Goiter	Negative	38	18	20	*P* > 0.05
	Positive	22	10	12

A. Relative levels of circ-CCDC66 in PTC specimens (n = 60) and paracancerous ones (n = 60) detected by qRT-PCR; B. Correlation between circ-CCDC66 level and AJCC staging of PTC; C. Correlation between circ-CCDC66 level and lymphatic metastasis of PTC; D. Kaplan–Meier curves depicted survival of PTC patients with high or low level of circ-CCDC66 (*p* = 0.0058); E. Relative levels of circ-CCDC66 in PTC cell lines detected by qRT-PCR; F. Relative levels of circ-CCDC66 and linear CCDC66 mRNA in K1 and TPC-1 cells exposed to Actinomycin D detected by qRT-PCR; G. Stability of circ-CCDC66 and linear CCDC66 mRNA in K1 and TPC-1 cells induced with RNase R. *p < 0.05; **p < 0.01.

### 3.2. Knockdown of circ-CCDC66 suppresses proliferative, migratory and invasive capacities of PTC cells

**Figure 2.**
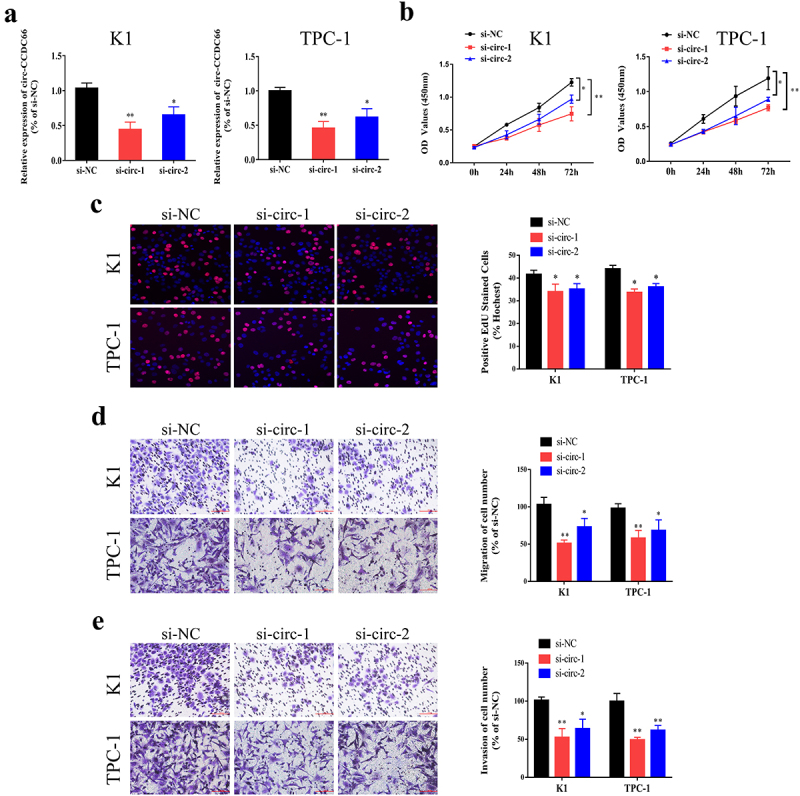
Knockdown of circ-CCDC66 suppressed proliferative, migratory and invasive capacities of PTC.

To explore the role of circ-CCDC66 in PTC, the expression of circ-CCDC66 was silenced by siRNA transfection, and the transfection efficacy of circ-CCDC66 siRNAs in K1 and TPC-1 cells was examined by qRT-PCR (Figure 2a). CCK-8 and EdU assays showed that knockdown of circ-CCDC66 in PTC cells markedly attenuated proliferative capacity (Figure 2b-c). Furthermore, the Transwell assay revealed that inhibition of circ-CCDC66 suppressed the migratory and invasive capacities of K1 and TPC-1 cells (Figure 2d-e). Taken together, these results suggest that circ-CCDC66 may play an oncogenic role in the development of PTC.

A. Transfection efficacy of circ-CCDC66 siRNAs in K1 and TPC-1 cells examined by qRT-PCR; B-C. Proliferative capacity of K1 and TPC-1 cells transfected with si-circ-CCDC66 or si-NC examined by CCK-8 and EdU assay; D-E. Migratory and invasive capacities of K1 and TPC-1 cells transfected with si-circ-CCDC66 or si-NC examined by Transwell assay. *p < 0.05; **p < 0.01.

### 3.3. Circ-CCDC66 can bind to miR-129-5p

**Figure 3.**
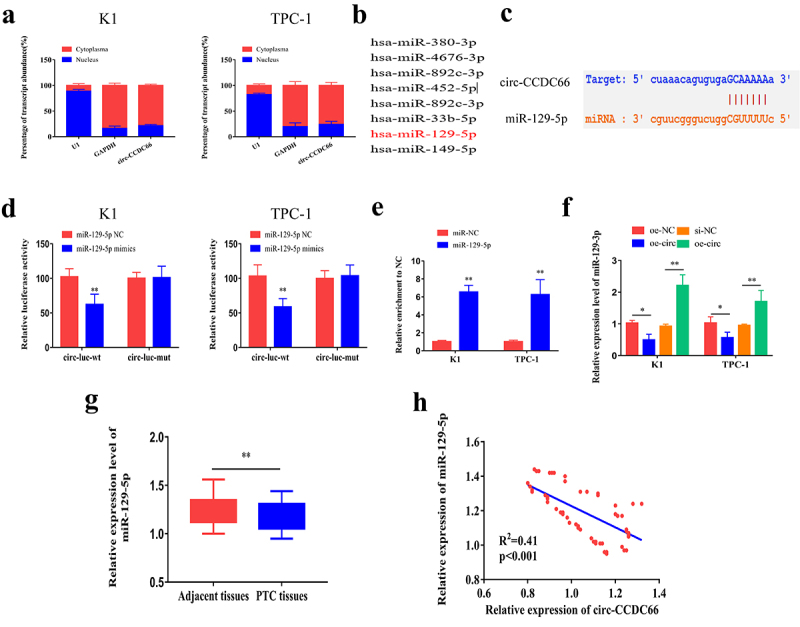
Circ-CCDC66 could bind miR-129-5p.

Furthermore, we investigated the mechanism through which circ-CCDC66 promotes malignant progression of PTC. First, we explored the subcellular distribution of circ-CCDC66. We observed that circ-CCDC66 was mainly distributed in the cytoplasm of PTC cells, suggesting a role of circ-CCDC66 in mediating post-transcriptional levels of genes (Figure 3a). By analyzing the Starbase database (http://starbase.sysu.edu.cn/), the following candidate miRNAs were identified to bind circ-CCDC66 in the promoter region: miR-129-5p, miRNA-380-3p, miRNA-4676-3p, miRNA-892c-3p, miRNA-452-5p, miRNA-33b-5p, and miRNA-149-5p (Figure 3b). Among the screened miRNAs, miR-129-5p was the most likely to bind circ-CCDC66. We constructed WT and MUT circ-CCDC66 vectors based on the binding sites of circ-CCDC66 and miR-129-5p (Figure 3c). Overexpression of miR-129-5p in K1 and TPC-1 cells markedly decreased luciferase activity in the WT circ-CCDC66 vector, while the luciferase activity in the MUT vector was not affected, confirming that circ-CCDC66 bound miR-129-5p (Figure 3d). Furthermore, the interaction between the two was also confirmed by RNA immunoprecipitation (RIP) (Figure 3e). We also confirmed that circ-CCDC66 could bind to miR-129-5p in 293 T cells by dual-luciferase reporter gene and RIP assays (Figure S1A–B). In K1 and TPC-1 cells, circ-CCDC66 negatively regulated expression of miR-129-5p (Figure 3f). Additionally, qRT-PCR data revealed that miR-129-5p expression in PTC specimens was low (Figure 3g). The expression level of miR-129-5p was also significantly associated with tumor size, TNM stage, and lymph node metastasis in patients with PTC (Table 3). miR-129-5p expression was found to be negatively correlated with circ-CCDC66 levels in PTC specimens (R^2^ = 0.41, *p* < 0.001) (Figure 3h). Through in vitro experiments, we found that overexpression of circ-CCDC66 might promote the EMT signaling, while overexpression of miR-129-5p could inhibit the EMT pathway (Figure S2). We speculated that circ-CCDC66 might affect the malignant progression of PTC by regulating the EMT pathway.

**Table 3.** Relationship between miR-129-3p expression and the clinical pathological characteristics of PTC patients (n = 60)

Clinic pathological features		NO. of cases	miR-129-3p (n, %)		*p* – value
			Low	High	
Gender	Male	32	16	16	*P* > 0.05
	Female	28	15	13	
Age	≤ 55	40	17	23	*P* > 0.05
	> 55	20	11	9	
Extra thyroidal extension	Negative	19	6	13	*P* > 0.05
	Positive	41	24	17	
Tumor size	≤1	42	15	27	*P* = 0.0463
	>1	18	12	6	
TNM stage	I/II	34	11	23	*P* = 0.0086
	III/IV	26	18	8	
Lymph node metastasis	Negative	27	10	17	*P* = 0.0185
	Positive	33	23	10	
Nodular Goiter	Negative	38	22	16	*P* > 0.05
	Positive	22	11	11

A. Subcellular distribution of circ-CCDC66 in K1 and TPC-1 cells; B. Potential miRNAs that could bind circ-CCDC66 as predicted using starBase (http://starbase.sysu.edu.cn/); C. Construction of wild-type and mutant-type circ-CCDC66 vectors based on the predicted binding sites of circ-CCDC66 and miR-129-5p in starBase (http://starbase.sysu.edu.cn/); D. Dual-luciferase reporter assay showed luciferase activity in wild-type and mutant-type circ-CCDC66 vectors transfected with either miR-129-5p mimics or NC; E. RIP showed the interaction between circ-CCDC66 and miR-129-5p; F. Relative levels of miR-129-5p in K1 and TPC-1 cells with overexpression or knockdown of circ-CCDC66; G. Relative levels of miR-129-5p in PTC and paracancerous specimens; H. Pearson’s correlation test obtained a negative correlation between circ-CCDC66 and miR-129-5p in PTC specimens (R^2^ = −0.41, *p* < 0.001). *p < 0.05; **p < 0.01.

## 3.4. miR-129-5p can bind to LARP1

**Figure 4.**
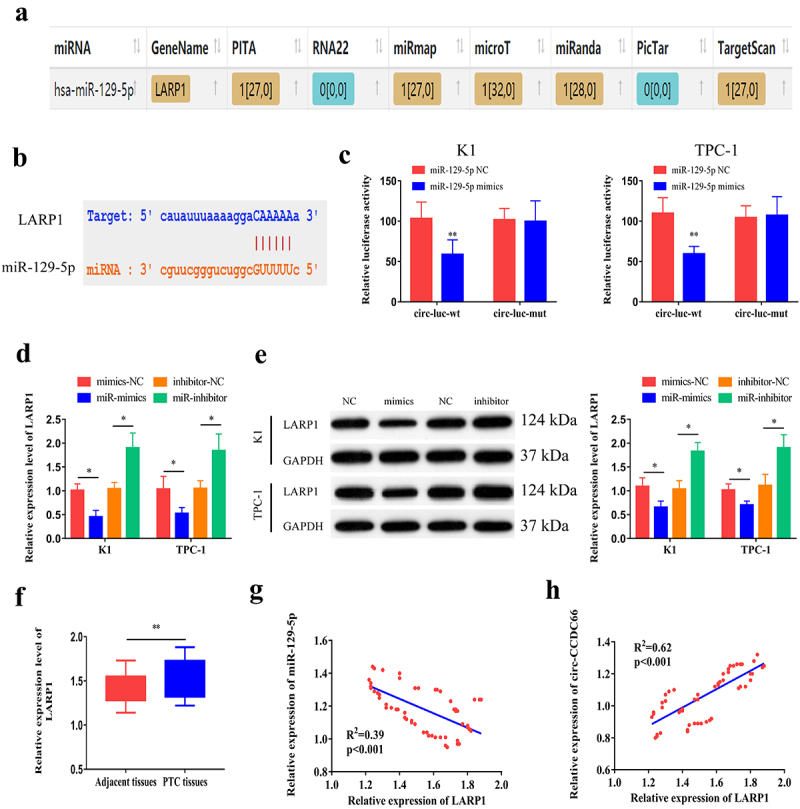
**miR-129-5p** could bind LARP1.

By analyzing microT, PITA, miRmap, microT, miRanda, and Targetscan databases, we searched for potential genes that could bind to miR-129-5p. After cross-matching the predicted data, *LARP1* was identified as a potential target (Figure 4a). We then constructed WT and MUT LARP1 vectors (Figure 4b) to examine the binding relationship between miR-129-5p and LARP1 using a dual-luciferase reporter assay (Figure 4c). In K1 and TPC-1 cells, overexpression of miR-129-5p downregulated the mRNA and protein levels of LARP1, while knockdown of miR-129-5p resulted in upregulation of LARP1 at the mRNA and protein levels (Figure 4d-e). Compared with paracancerous specimens, LARP1 was highly expressed in PTC tissues (Figure 4f). In addition, the expression level of LARP1 was significantly associated with tumor size, TNM stage, and lymph node metastasis in patients with PTC (Table 4). Moreover, we also performed IHC to examine LARP1 protein expression in normal and PTC tissues. We observed that LARP1 protein expression levels were higher in PTC tissues than in normal tissues (Figure S3A). Kaplan–Meier survival analysis from our data and The Cancer Genome Atlas (TCGA) dataset (http:// www.oncolnc.org/) revealed that LARP1 expression levels were not significantly correlated with the prognosis of PTC patients (Figure S3B–C). However, LARP1 expression was negatively correlated with miR-129-5p levels (R^2^ = 0.39, *p* < 0.001) and positively correlated with circ-CCDC66 levels in PTC specimens (R^2^ = 0.62, *p* < 0.001) (Figure 4g-h). Taken together, these results suggest that, circ-CCDC66 competitively binds to miR-129-5p to upregulate LARP1 expression.

**Table 4.** Relationship between LARP1 expression and the clinical pathological characteristics of PTC patients (n = 60)

Clinic pathological features		NO. of cases	LARP1 (n, %)		*p* – value
			Low	High	
Gender	Male	32	14	18	*P* > 0.05
	Female	28	12	16	
Age	≤ 55	40	16	24	*P* > 0.05
	> 55	20	9	11	
Extra thyroidal extension	Negative	19	10	9	*P* > 0.05
	Positive	41	18	23	
Tumor size	≤1	42	25	17	*P* = 0.0112
	>1	18	4	14	
TNM stage	I/II	34	20	14	*P* = 0.0392
	III/IV	26	8	18	
Lymph node metastasis	Negative	27	20	7	*P* = 0.0046
	Positive	33	12	21	
Nodular Goiter	Negative	38	20	18	*P* > 0.05
	Positive	22	9	13

A. Potential targets of miR-129-5p predicted in microT, PITA, miRmap, microT, miRanda and Targetscan, and LARP1 was screened out after cross-match; B. Construction of wild-type and mutant-type LARP1 vectors based on the predicted binding sites of LARP1 and miR-129-5p in starBase (http://starbase.sysu.edu.cn/); C. Dual-luciferase reporter assay showed luciferase activity in wild-type and mutant-type LARP1 vectors transfected with either miR-129-5p mimics or NC; D-E. The mRNA and protein levels of LARP1 in K1 and TPC-1 cells with overexpression or knockdown of miR-129-5p; F. Relative levels of LARP1 in PTC and paracancerous specimens; G. Pearson’s correlation test obtained a negative correlation between LARP1 and miR-129-5p in PTC specimens (R^2^ = 0.39, *p* < 0.001); H. Pearson’s correlation test obtained a positive correlation between LARP1 and circ-CCDC66 in PTC specimens (R^2^ = 0.62, *p* < 0.001). *p < 0.05; **p < 0.01.

### 3.5. Circ-CCDC66 is involved in the development of PTC through the miR-129-5p/LARP1 axis

**Figure 5.**
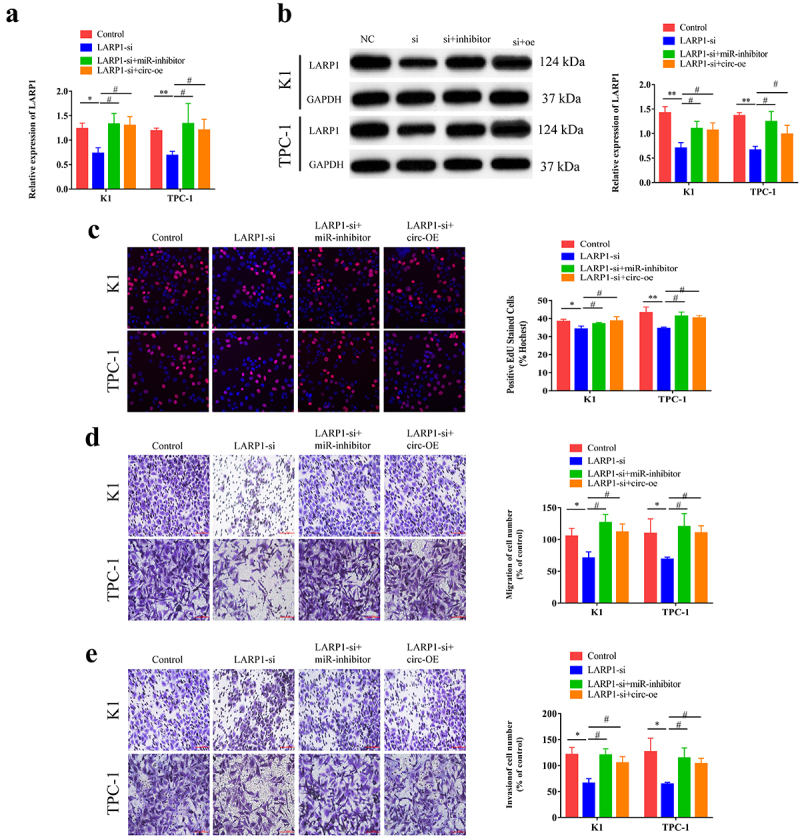
Circ-CCDC66 was involved in the development of PTC through the miR-129-5p/LARP1 axis.

To ascertain the role of the circ-CCDC66/miR-129-5p/LARP1 axis in the development of PTC, we co-transfected LARP1-siRNA, or miR-129-5p inhibitor and circ-CCDC66-OE in K1 and TPC-1 cells. Higher levels of LARP1 were observed in cells co-transfected with LARP1-siRNA and circ-CCDC66-OE, or LARP1-siRNA and miR-129-5p inhibitor than in cells transfected with LARP1-siRNA alone (Figure 5a-b). Subsequently, we examined the influence of the circ-CCDC66/miR-129-5p/LARP1 axis on PTC cell behavior. LARP1 knockdown attenuated the proliferative, migratory, and invasive capacities of PTC cells. These effects were partially relieved by co-knockdown of LARP1 and miR-129-5p, or co-transfection of LARP1-siRNA and circ-CCDC66-OE (Figure 5c-e). These results suggested that circ-CCDC66 competitively binds miR-129-5p to upregulate LARP1, thus promoting the proliferative, migratory, and invasive capacities of PTC cells.

A. Relative level of LARP1 in K1 and TPC-1 cells co-transfected with LARP1-siRNA or miR-129-5p inhibitor and circ-CCDC66-OE; B. Protein level of LARP1 in K1 and TPC-1 cells co-transfected with LARP1-siRNA or miR-129-5p inhibitor and circ-CCDC66-OE; C. Proliferative capacity of K1 and TPC-1 cells co-transfected with LARP1-siRNA or miR-129-5p inhibitor and circ-CCDC66-OE examined by EdU assay; D-E. Migratory and invasive capacities of K1 and TPC-1 cells co-transfected with LARP1-siRNA or miR-129-5p inhibitor and circ-CCDC66-OE. *p < 0.05; **p < 0.01; #*p* < 0.05.

### 3.6. Inhibition of circ-CCDC66 can inhibit the growth of PTC tumor

**Figure 6.**
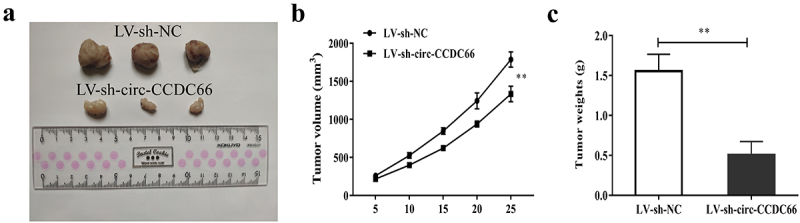
Inhibition of circ-CCDC66 could suppress PTC tumor growth.

We also explored the effect of circ-CCDC66 inhibition on PTC tumor growth in a mouse xenograft model. As shown in Figure 6a-c, after 25 days of cancer cell inoculation, the tumor growth volume and weight of nude mice injected with PTC cells transfected with LV-sh-circ-CCDC66 were significantly smaller than those of LV-sh-NC.

A. Nude mouse models were established via subcutaneous injection of TPC-1 cells and divided into LV-sh-NC group and LV-sh-circ-CCDC66 group; B-C. Effects of sh-circ-CCDC66 on tumor volume and tumor weight. **p < 0.01.

## 4. Discussion

CircRNAs can act as ceRNAs that bind to miRNAs and participate in the progression and development of various cancers [53,54]. Cancer is a critical disease that threatens human lives worldwide and has been highlighted in life science research because of its complicated pathogenesis and limited therapeutic strategies. Many studies have shown that circRNAs are involved in cancer development and are promising novel biomarkers and therapeutic targets. The biological functions of circRNAs in the development of PTC have been reported previously. It has been reported that circ_PSD3 can stimulate the progression of PTC by modulating the miRNA-637/HEMGN axis and activating the PI3K/Akt signaling pathway [55]. CircEIF3I can competitively bind to miRNA-149 to upregulate KIF2A, thus accelerating the development of PTC [56]. The hsa_circ_0058124/NOTCH3/GATAD2A regulatory axis is critical to the carcinogenesis of PTC and its invasiveness and is a promising target for the management of PTC [57]. Our study revealed that circ-CCDC66 is upregulated in PTC specimens and correlates with poor clinical characteristics in PTC cases. *In vitro* experiments also demonstrated the oncogenic role of circ-CCDC66 in modulating the proliferative, migratory, and invasive capacities of PTC cells.

We investigated the mechanism through which circ-CCDC66 regulates PTC cell functions. Our results suggest that circ-CCDC66 is able to bind to miR-129-5p and inhibit its expression. miR-129-5p expression was also found to be markedly downregulated in PTC specimens. miR-129-5p is a functional miRNA involved in several types of tumors, including breast cancer [58], glioblastoma [59], gastric cancer [60,61], lung cancer [62], bladder cancer [63] and prostate cancer [64]. Zhang et al. [65] hypothesized that lncRNA NEAT1 participates in the development of PTC through the miR-129-5p/KLK7 axis.

By analyzing online bioinformatics databases, we identified *LARP1* as a potential target gene of miR-129-5p. LARP1 is an RNA-binding protein that was first discovered in *Drosophila melanogaster*. LARP1 has vital biological functions, especially in embryogenesis and cell cycle progression [66,67]. Members of the LARP family include LARP1, LARP1b, LARP4, LARP4b, LARP6, and LARP7, which are important for mRNA transcription or translation [66,67]. *LARP1* is located on human chromosome 5 and encodes a protein containing 1,096 amino acids. LARP1 lacks an enzymatic domain, but the presence of LA structure combined with an RNA recognition motif, as well as a highly conserved DM15 domain is attributed to the function of LARP1 in protein translation and mRNA translation, respectively, via recognition of certain nuclear fragments [68,69]. So far, the function of the DM15 domain is inconsistent, which may be associated with the nuclear acid regulation and gene translation that are responsible for carcinogenesis. LARP1 is of significance in osteosarcoma [70], prostate cancer [71], non-small cell lung cancer [72], CRC [73] and ovarian cancer [74]. However, the role of LARP1 in PTC requires further investigation.

Furthermore, we conducted a dual-luciferase reporter, RIP and Western blot assays, and found that miR-129-5p could target LARP1, while circ-CCDC66 promotes LARP1 expression by competitively binding to miR-129-5p. In addition, knockdown of LARP1 suppressed the proliferative, migratory, and invasive capacities of PTC cells as well as PTC tumor growth in a mouse xenograft model. Moreover, the oncogenic function of LARP1 could be partially reversed by knockdown of miR-129-5p or overexpression of circ-CCDC66.

Nevertheless, the present study has several limitations. First, we did not screen the differentially expressed circRNAs in PTC tissues using high-throughput sequencing technology, and the study lacked innovation. Second, circ-CCDC66 can bind to many miRNAs. We only investigated miR-129-5p, which has the strongest binding ability. Whether circ-CCDC66 can coordinately regulate the expression of LARP1 by binding to other miRNAs remains to be investigated. In addition, cell proliferation, migration, and invasion involve many related pathways, and whether circ-CCDC66 can regulate the biological effects of tumor cells by regulating the expression of these pathways remains to be explored further.

## 5. Conclusion

In conclusion, this study demonstrated that circ-CCDC66 was upregulated in PTC tissues and correlated with the poor prognosis in PTC patients. Circ-CCDC66 was also found to promote PTC cell proliferation, migration, and invasion by modulating the miR-129-5p/LARP1 regulatory axis. The circ-CCDC66/miR-129-5p/LARP1 axis may be a promising target for prognosis and therapy of PTC.

## Supplementary Material

Supplemental Material

## Data Availability

All data included in this study are available upon request by contacting the corresponding author.
