# Supplementary figures and images for: Circular RNA coiled-coil domain containing 66 regulates malignant development of papillary thyroid carcinoma by upregulating La ribonucleoprotein 1 via the sponge effect on miR-129-5p
Source: Bioengineered. 2022 Mar 9;13(3):7181–96. doi: 10.1080/21655979.2022.2036304 (PMC8973727; doi:10.1080/21655979.2022.2036304)

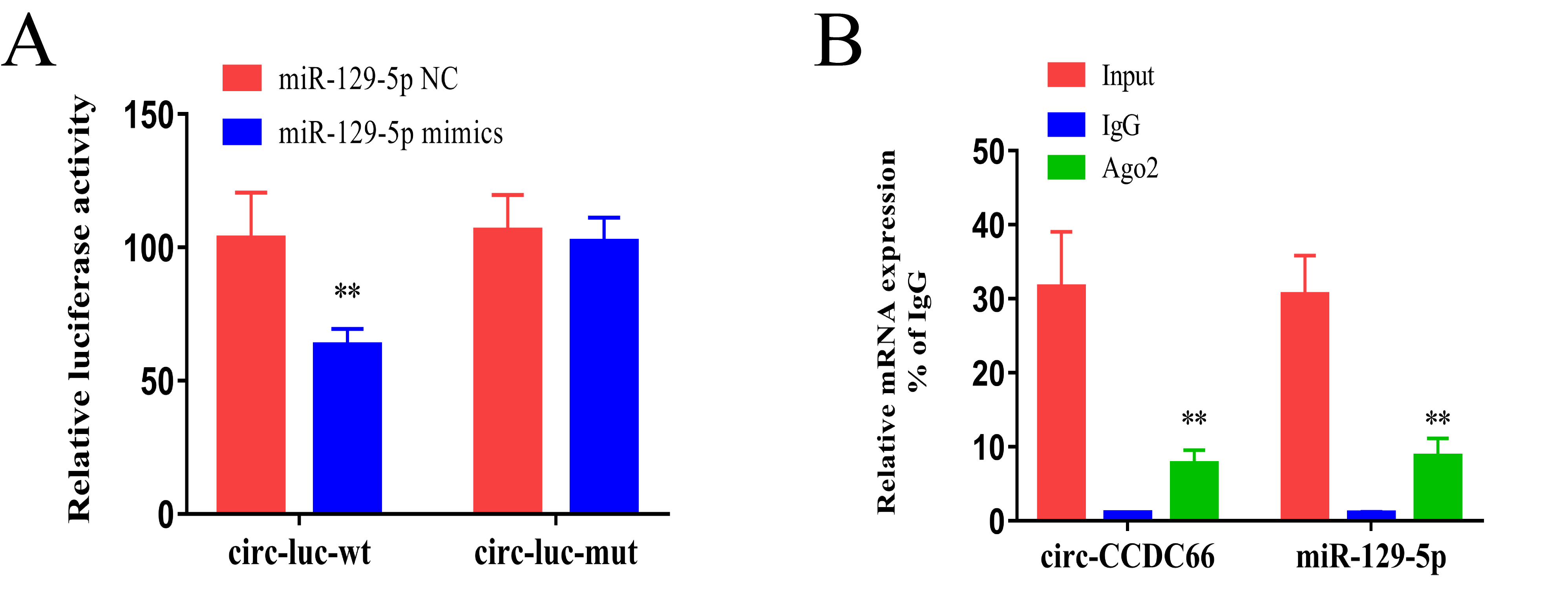

Supplement: Supplemental Material [file KBIE_A_2036304_SM9589.zip › supplementary/Figure S1.tif]

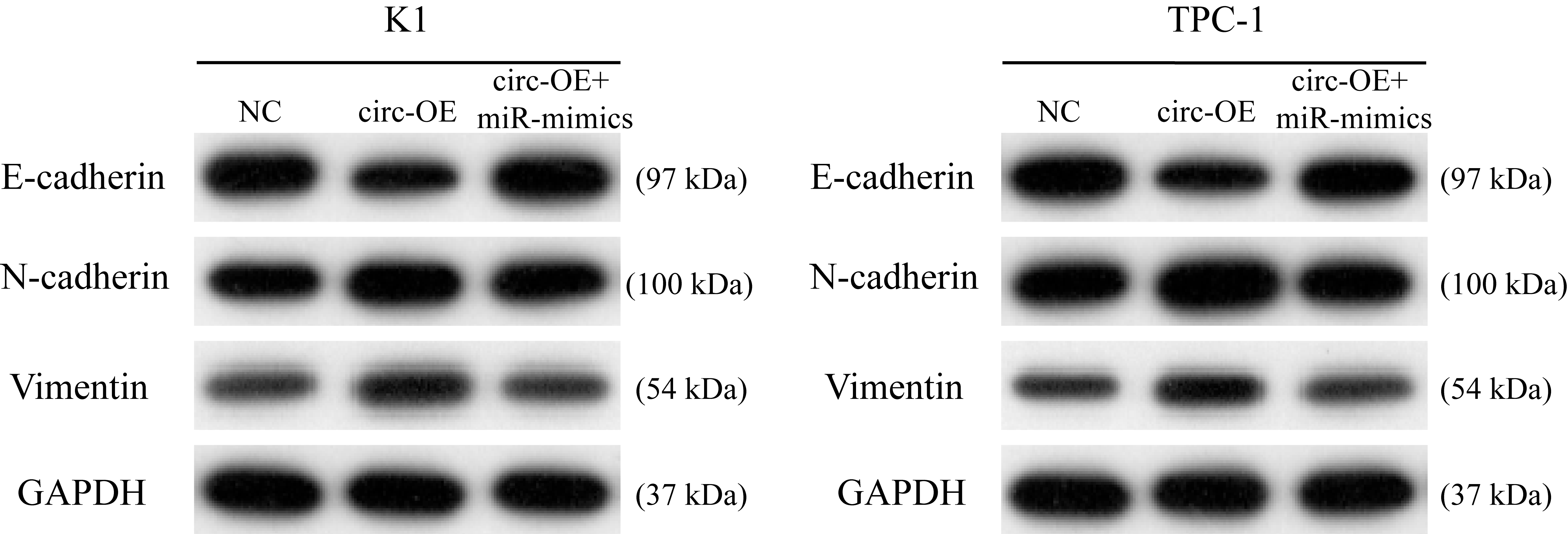

Supplement: Supplemental Material [file KBIE_A_2036304_SM9589.zip › supplementary/Figure S2.tif]

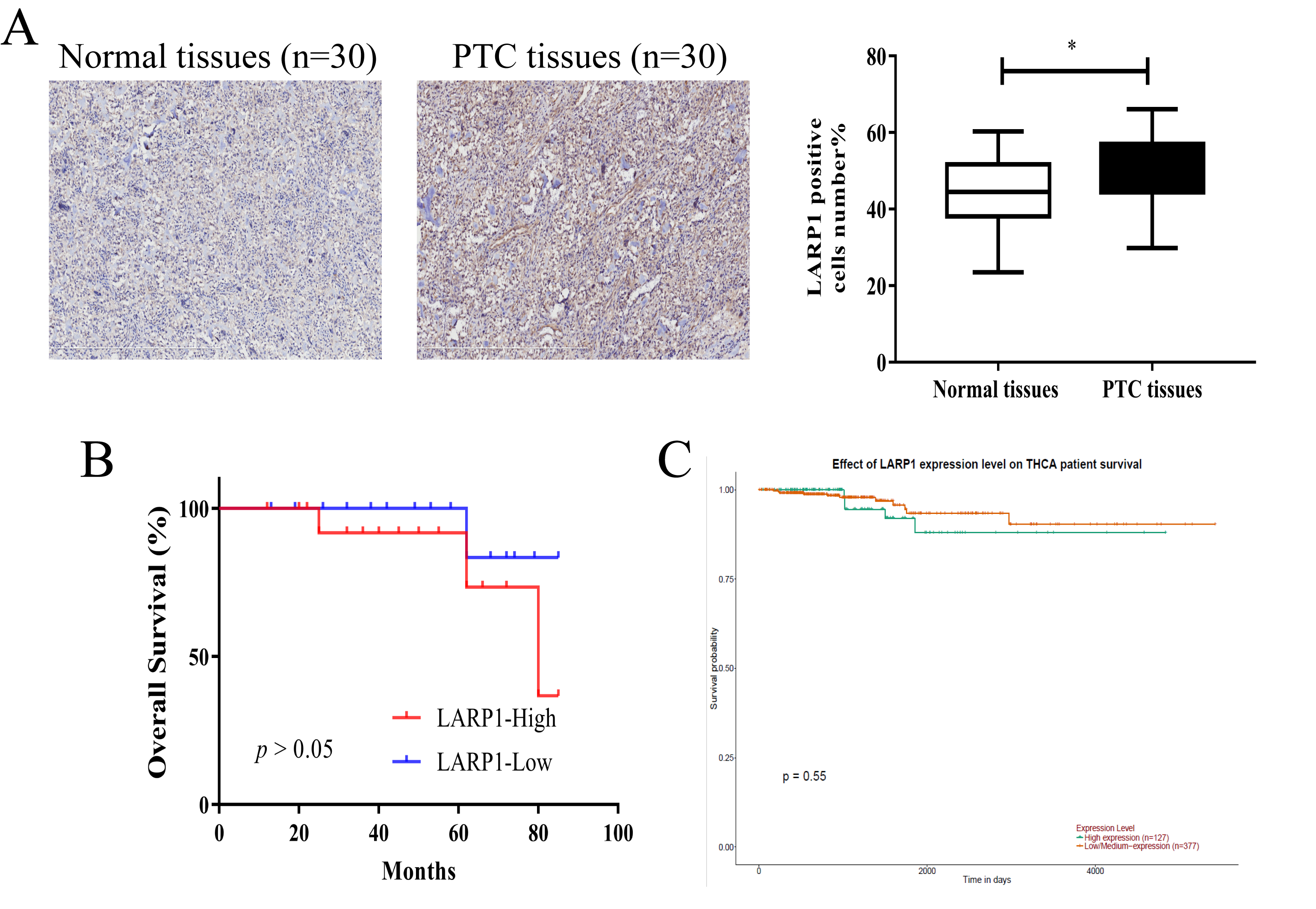

Supplement: Supplemental Material [file KBIE_A_2036304_SM9589.zip › supplementary/Figure S3.tif]
